# Medical Products and Environmentally Friendly Purchase Intention: What Is the Role of Green Consumers Behavior, Environment Concern, and Recycle Behavior?

**DOI:** 10.3389/fpubh.2022.960654

**Published:** 2022-07-22

**Authors:** Fachrurazi Fachrurazi, Fajar Purwanto, Dwi Dewianawati, Bambang Purwoko, Didit Darmawan

**Affiliations:** ^1^Faculty of Islamic Economic and Business, IAIN Pontianak, Pontianak, Indonesia; ^2^Department of Management, Universitas Mayjen Sungkono, Mojokerto, Indonesia; ^3^Department of Management, Universitas WR Supratman, Surabaya, Indonesia; ^4^Department of Management, Universitas Sunan Giri, Sidoarjo, Indonesia

**Keywords:** green consumers behavior, concern environment, recycle behavior, purchase intention, medical products, eco-friendly

## Introduction

According to Gan et al. (1), in the digital era and globalization, environmental preservation is a priority for various countries in the world, including Indonesia. Sustainable development is a conscious and planned effort that combines aspects of the social and economic environment in a development strategy to ensure the integrity of the environment, the safety of welfare capabilities, and the quality of life of present and future generations. The program is an effort to improve the quality of life of the community, while still paying attention to environmental sustainability. Sustainable development can be successful with the support of all components of society. Efforts that have been made by the government and the community, in the field of business, and efforts to preserve the environment are realized by implementing go green in various fields, namely marketing, production, and finance. In the household sector, consumers are known as green consumers. According to Pruša and Sadílek (2), green consumers are defined as individuals who make purchases by first thinking about the impact on the environment of the goods they consume. According to Yunarsih et al. (3), the behavior of consumers who care about the environment will be motivated to consume environmentally friendly products. Behavior based on concern for the environment or green consumer behavior is reflected by individual behavior when looking for, using, evaluating, and disposing of products, while product purchase decisions by consumers are often based on their attitude to the environment (environmental attitude). Attitude is one of the factors that affect environmental quality and is the most consistent explanatory factor in predicting consumer readiness to pay for environmentally friendly products.

Environmental problems have been a concern of the world community since several years ago, especially those related to waste. According to Makhdoomi and Nazir (4) and Noor et al. (5) Efforts to reduce the environmental impact of solid waste can be carried out by the country by minimizing the use of plastic, namely the restriction of plastic products that are highly used to reduce marine and land waste for its member countries. Environmental damage is also caused by rapid growth and encourages an economy that consumes and over-exploits natural resources. According to Gan et al. (1) and Langinier and Chaudhuri (6), the decline in the quality of social life and health is the impact of environmental damage, in addition to the impact of global warming, environmental degradation, and depletion of the ozone layer. According to Makhdoomi and Nazir (4), Langinier and Chaudhuri (6), and Lemke and Luzio (7), the growth of movements, such as Earth Day, going to work using a bicycle instead of a car, and several movement activities that support environmental conservation and a healthier lifestyle. The ratification of education on environmental sustainability is increasing, as well as increasing people's purchasing power, making Indonesia a potential market. Although Indonesia has great potential, there is still relatively little information about green consumer behavior compared to developed countries, before starting the environmental care movement. The description of the background shows that there is a relationship between consumer green behavior, knowledge, attitudes, behavior cycle repetition, and political action. In particular, this study aims to examine the effect of environmental knowledge attitudes on environmental behavior, and political action on green consumer behavior. In the long term, this research can contribute to the solution of the problem of environmental sustainability based on the idea that people who are aware of purchasing behavior will be able to play a role in overcoming environmental problems.

According to Makhdoomi and Nazir (4) and Lemke and Luzio (7), green consumer behavior is the behavior of individuals who are influenced by their concern for the environment. This behavior is reflected by the individual, when he/she searches for, buys, uses, evaluates, and disposes products. According to Nyborg et al. (8) and Pillai (9), consumers with a high level of environmental awareness make increased purchasing decisions for environmentally friendly products compared to products that pay less attention to environmental issues. Thus, awareness of environmental measures will be more closely related to buying habits than sociodemographic or personality variables. According to Zhao et al. (10) and Ziaei Bide and Hosseini (11), to see the level of consumer awareness of environmental sustainability, their purchasing behavior toward environmentally friendly products can explain it. Insights and consumer knowledge became important factors in efforts to achieve go green in Indonesia. The public knowledge to preserve the environment is still relatively low, so it needs serious attention. According to Gan et al. (1), Langinier and Chaudhuri (6), and Lemke and Luzio (7), low consumer insight and knowledge about the environment have an impact on green marketing activities (marketing with environmental insight), which are still few, and consumer pro-environment behavior is still relatively low in Indonesia. Consumer awareness will emerge and grow strong if provided with complete and accurate information and knowledge about environmental issues. According to Pruša and Sadílek (2), Nyborg et al. (8), Pillai (9), and Park and Lee (12), good customer knowledge will encourage positive behavior toward environmental sustainability. The higher the level of public knowledge about the environment, the more awareness to buy environmentally friendly products. Therefore, manufacturers need to implement strategies, including creating and using environmentally friendly components, installing environmentally friendly labels to standardize products, and certifying and communicating that the products they offer are classified as environmentally friendly products.

According to Chen et al. (13) and Farzin et al. (14), environmental care attitude is an evaluation of the form of feelings and potential tendencies to react, which is the result of the interaction between cognitive, affective, and conative components of the environment to be one of the factors in efforts to improve environmental quality. This can be interpreted that the concern for the environment is positively related to attitudes. According to Farzin et al. (14), seven variables influence a person's environmental behavior, one of which is an environmental attitude, which refers to an individual's cognitive assessment to mark a protective environment. Attitude toward the environment refers to an individual's marking consideration toward environmental protection.

Increasing public awareness of environmental sustainability can be done through continuous socialization about the consequences of ignoring the environment. According to Farzin et al. (14), recycling is a process to turn used goods into new goods to prevent scattered waste. Cycle repeat is a solid waste management strategy consisting of waste sorting activities that can be recycled, reworked, collected, further processed, and distributed, as well as the manufacturing of used products/materials, and main components. According to Agyeman (15) and Ahmadi et al. (16), recycling behavior is the behavior of individuals who are influenced by their concern for the environment, which is reflected in the way individuals prevent the presence of waste through solid waste management. A program cycle that repeats itself will only succeed if it is actively supported by the community participating in it. This is in line with the research results of Chen et al. (13), Farzin et al. (14), and Ahmadi et al. (16), which showed that there is a positive relationship between concern for the environment and recycling repeat behavior.

## Results, Discussion, and Opinion

**Figure 1:**
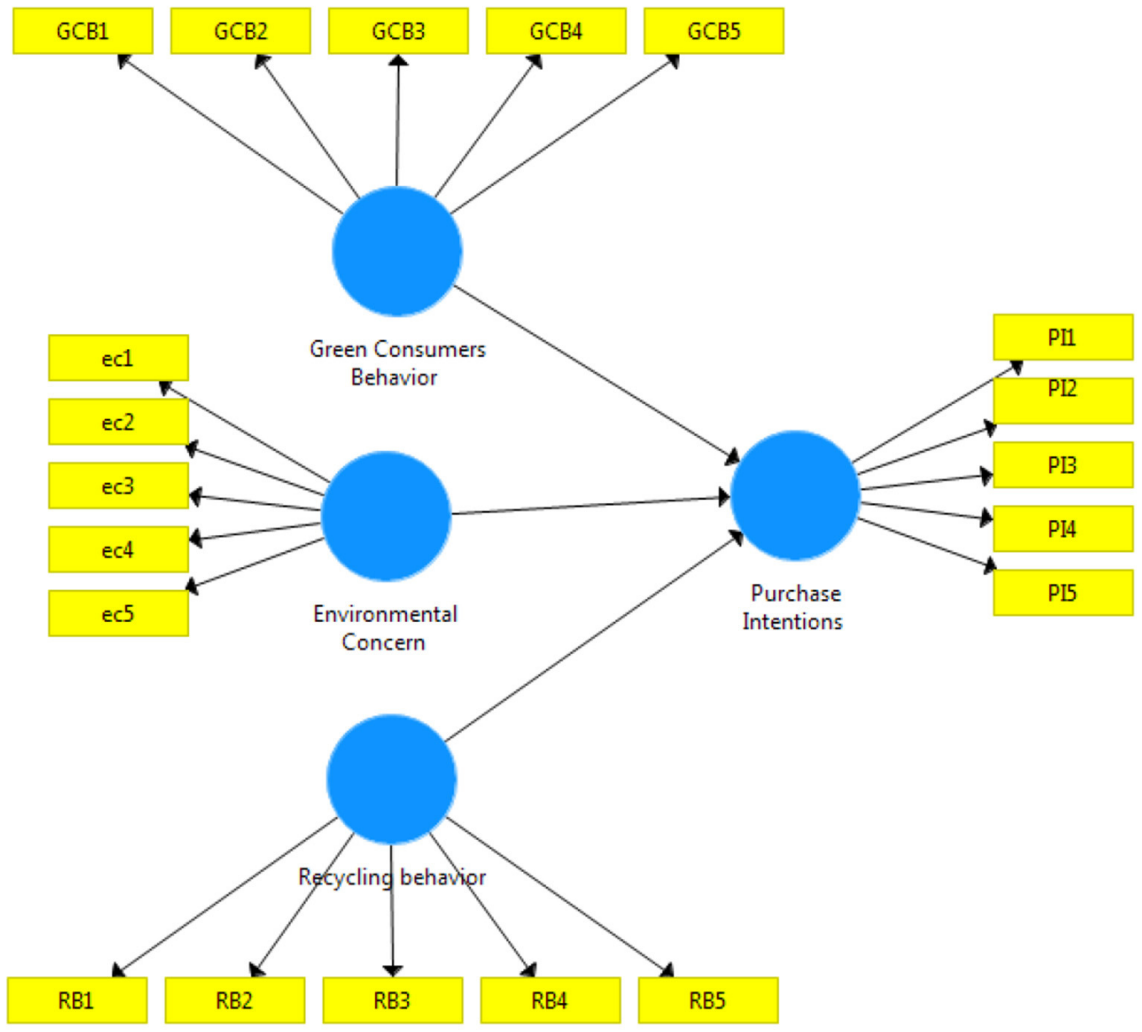
Conceptual model.

According to Figure 1 green consumer behavior has a significant effect on medical purchase intention, the variable green consumer behavior improvement will be followed by a significant increase in the purchase intention variable. This is in line with the research of Chen et al. (13), Farzin et al. (14), Agyeman (15), and Ahmadi et al. (16) that green consumer behavior has a significant effect on purchase intention.

Environmental concern has a significant effect on medical purchase intention; the variable increasing environmental concern will be followed by a significant increase in the purchase intention variable. This is in line with research by Pruša and Sadílek (2), Noor et al. (5), Nyborg et al. (8), Pillai (9), and Park and Lee (12) that environmental concern has a significant effect on purchase intention. Recycling behavior has a significant effect on medical purchase intention; the variable increased recycling behavior would be followed by a significant increase in purchase intention. This is in line with the research of Pruša and Sadílek (2), Pillai (9), and Park and Lee (12) that recycling behavior has a significant effect on purchase intention.

According to Permana and Soediantono (17) green human behavior in human relations with the environment requires environmental awareness and concern. The tendency to care for the environment continues to grow, along with increasing global warming. Environmental issues are generally related to the future. That is, pro-environmental actions and green behavior are expected to have long-term, not short-term effects. The millennial generation is a generation that is more future-oriented, so they are willing to sacrifice momentary satisfaction to achieve better long-term goals. According to Quddus et al. (18), millennials invest effort and resources in current activities with far-reaching returns and are willing to endure current unfavorable situations with the potential to lead to a positive future. The green image of their company is separate from certain products that are not environmentally safe and the company's operational activities are environmentally friendly. In addition to being environmentally friendly, consumers expect additional benefits from environmentally friendly products, such as cost savings or increased efficiency. Regarding suggestions based on the importance of future orientation and the benefits of eco-products, it is hoped that companies or business actors in Indonesia will always create product innovations based on environment friendly (19–21). A product to balance the needs of the millennial generation who think more about the long-term impact on the issue of climate change is increasingly getting the attention of many parties.

## Conclusion

Based on the results of data analysis, it is concluded that green consumer behavior has a significant effect on purchase intention, environmental care has a significant effect on purchase intention, and consumer recycling behavior has a significant effect on medical purchase intention of environmentally friendly products. This is because, at this moment, the public is more interested in applicable things that motivate and inspire them. Several examples of repeated cycles of behavior and pro-environmental political actions are tangible evidence that can motivate people to engage in green behaviors, including product buying behavior. However, simultaneously, environmental knowledge, environmental attitudes, and recycling behavior have a significant effect on the green consumer behavior variable. From these findings, efforts to improve recycling behavior can be carried out through the management of recycling programs in an open, sustainable manner, and intensively disseminated to the wider community through electronics to increase green consumer behavior. The repetition of the cyclical program can be carried out in the form of using or re-utilizing for other purposes, separating waste between those that can be recycled and those that cannot, and utilizing used goods for activities that have economic value. Efforts to increase political action can be done by establishing discussion forums on environmental concerns, both formal and informal, sharing information with friends and relatives regarding environmentally friendly products, and writing and speaking in discussion forums and mass media about the importance of green behavior. For companies, to increase millennial consumers' buying interest in environmentally friendly products, companies can increase consumer awareness of environmental problems because environmental care is the most influential variable in generating consumer buying interest in Indonesian millennial consumers. This can be done by the company by making advertisements that create awareness about the current environmental damage, the impact if the damage continues, and the ways to preserve the environment; one of which is by choosing environmentally friendly products. This method will make consumers more aware of environmental issues and create a positive perception in consumers' minds about environmentally friendly products.

## Author Contributions

All authors listed have made a substantial, direct, and intellectual contribution to the work and approved it for publication.

## Conflict of Interest

The authors declare that the research was conducted in the absence of any commercial or financial relationships that could be construed as a potential conflict of interest.

## Publisher's Note

All claims expressed in this article are solely those of the authors and do not necessarily represent those of their affiliated organizations, or those of the publisher, the editors and the reviewers. Any product that may be evaluated in this article, or claim that may be made by its manufacturer, is not guaranteed or endorsed by the publisher.
